# Comparison of clinical efficacy of sequential and single intra-articular injection of platelet-rich plasma in treatment of early/mid-stage knee osteoarthritis

**DOI:** 10.3389/fmed.2025.1707979

**Published:** 2025-12-11

**Authors:** Wei Zhao, Lin Chen, Deli Wang, Xiuli Wei

**Affiliations:** Department of Rehabilitation Medicine, Renmin Hospital, Hubei University of Medicine, Shiyan, Hubei, China

**Keywords:** knee osteoarthritis, platelet-rich plasma, intra-articular injection, inflammatory responses, extracellular matrix

## Abstract

**Objective:**

To evaluate the clinical efficacy of sequential and single injection of platelet-rich plasma (PRP) in treatment of early/mid-stage knee osteoarthritis (KOA).

**Methods:**

Ninety-four patients who were diagnosed of early/mid-stage KOA from 2022 to 2024 were included in this study, involving 39 case undergoing sequential intra-articular PRP injection (sequential PRP group) and 55 case undergoing single intra-articular PRP injection (single PRP group). Outcomes including serum interleukin-1β (IL-1β), interleukin-6 (IL-6), and tumor necrosis factor-α (TNF-α) levels, synovial fluid matrix metalloproteinase-3 (MMP-3) and tissue inhibitor of metalloproteinases-1 (TIMP-1) levels, visual analog scale (VAS) score, Western Ontario and McMaster Universities Arthritis Index (WOMAC) score, Lysholm score and complications were all recorded and compared.

**Results:**

At 6-month post-treatment, not only serum IL-1β, IL-6, and TNF-α, but also synovial fluid MMP-3 and TIMP-1, were significantly improved compared with those pre-treatment in each group. Compared with single PRP group, however, the sequential PRP group showed lower serum IL-1β, IL-6, TNF-α, synovial fluid MMP-3 and higher synovial fluid TIMP-1 at 6-month post-treatment. Both the two groups achieved significant improvements in VAS score, WOMAC score and Lysholm score at 1, 3, and 6 months post-treatment. Although no significant differences were found in VAS score, WOMAC score and Lysholm score between single PRP group and sequential PRP group at 1 and 3 months post-treatment, sequential PRP group had a lower VAS score and WOMAC score, and a higher Lysholm score at 6 months post-treatment. No significant difference was found in complications rate between the two groups and all cases were cured after active treatment.

**Conclusions:**

Both sequential and single injection of PRP can achieve satisfactory clinical efficacy in the treatment of early/mid-stage KOA, but sequential intra-articular PRP injection has the advantages of sustained long-term efficacy in relieving pain and improving knee joint function.

## Introduction

Knee osteoarthritis (KOA) is a common degenerative cartilage disorder associated with structural and functional changes in the knee joint (1). Patients with KOA typically experience symptoms such as joint pain, stiffness, limited mobility, and functional impairment, which significantly reduce their quality of life and impose substantial burdens on both individuals and families (1). The knee, being one of the body's most weight-bearing joints, is particularly susceptible to structural damage from aging, wear, trauma, and developmental abnormalities, ultimately leading to KOA (2). It is reported that approximately 250 million people worldwide are affected by KOA, making it a major global public health concern (3).

The primary treatment goals for KOA are to alleviate pain and improve joint function (4). Management of KOA involves both pharmacological treatment and surgical interventions. Surgery, such as knee arthroplasty, is considered an effective therapy, primarily indicated for advanced-stage KOA patients (5). Therefore, conservative treatment remains the first choice for early-stage KOA patients (6). Nonsteroidal anti-inflammatory drugs (NSAIDs), while being primary treatment options for KOA, may cause gastrointestinal, cardiovascular, and renal adverse events due to systemic administration (6). Intra-articular injection therapy has gained increasing attention for its direct targeting of affected tissues and reduced adverse reactions (7).

Platelet-rich plasma (PRP) is a blood plasma derived from autologous blood, which contains high concentrations of platelets and growth factors, and acts as a reservoir for cytokines, including growth factors, chemokines, and various mediators (8). These growth factors can activate local stem cells and fibroblasts, mobilize them to damaged areas, stimulate angiogenesis in affected regions, control inflammation, block catabolism and cytokine production, and also prompt neighboring healthy cells to secrete additional growth factors (8). Studies have shown that the direct application of PRP to cartilage injury sites can effectively promote healing processes and accelerate the formation of repaired cartilage tissue (9). In recent years, intra-articular PRP injections for the treatment of KOA have gained increasing attention among orthopedic surgeons and researchers (10). It has been reported that intra-articular PRP injection therapy can help KOA patients resume movement and activities earlier by alleviating pain and improving endurance within 2–3 weeks (11). However, the optimal PRP injection dosage and frequency for KOA patients remain controversial (12).

Therefore, we conducted this retrospective single-center cohort study to compare the clinical efficacy of sequential and single intra-articular injections of PRP in the treatment of KOA.

## Materials and methods

All of the participants provided their written informed consent to participate in this study before their data were stored in the hospital database and used for research purposes. The work has been reported in line with the STROCSS criteria (13).

### Patients selection

Medical records of hospitalized patients diagnosed with KOA in our department from 2022 to 2024 were retrospective analyzed.

Inclusion criteria: (1) The symptoms were recurrent chronic pain or swelling of the knee joint, and X-ray showed degenerative changes in the knee joint, subchondral bone sclerosis and/or cystic changes, and osteophytes at the edge of the joint, which were consistent with the diagnosis of KOA. (2) According to the K-L grading criteria, the imaging results were in line with the grade I-III (14). (3) Patients were between 40 and 80 years old with unilateral KOA. (4) No history of using NSAIDs, glucocorticoid (GCs) drugs or drugs affecting coagulation within 4 weeks. (5) First use of PRP, and no history of using sodium hyaluronate (HA) within 6 months. (6) The follow-up time was more than 6 months. (7) The clinical and imaging data during the follow-up were complete.

Exclusion criteria: (1) History of trauma and surgery to the knee joint. (2) Consciousness and mental disorders. (3) Malignant tumors. (4) Severe cardiovascular and cerebrovascular diseases, liver, kidney, lung, and other organ dysfunction. (5) Coagulation disorders and platelet dysfunction diseases. (6) Women who were pregnant or breastfeeding. (7) Patients suffering from local or systemic infectious diseases, contagious diseases.

### Preparation of PRP

The nurse extracted 20 mL of venous blood from the patient's median cubital vein, including 1 mL of low molecular weight heparin sodium, and subsequently placed the sample in a centrifuge for processing. Following the initial centrifugation, the blood separated into three distinct layers: the top layer was plasma, the middle layer consisted of blood sediment, and the bottom layer was composed of red blood cells. The operator then used a syringe to extract the plasma layer and subjected it to a second round of centrifugation. After the second centrifugation, platelet-poor plasma was found at the top, while platelet-rich plasma (PRP) formed at the bottom. Upon removal of the top layer, approximately 3 mL of PRP was collected. The PRP administered to the patient was prepared using a centrifugation-based PRP preparation device (WEGO, China), and the preparation process was meticulously conducted by the same medical team, adhering strictly to operational standards and sterile procedures throughout.

### Intra-articular PRP injection method

The PRP was injected into the pathological joint cavity of KOA patients under ultrasound guidance, and the entire process strictly adhered to aseptic operation principles. The patient was positioned flat on the treatment bed with the knee joint to be injected slightly flexed for complete exposure. The operator conducted several passive flexion-extension movements of the knee joint. After disinfecting with iodophor, sterile gloves were donned and a sterile drape was laid out. Local infiltration anesthesia using 2% lidocaine hydrochloride was administered to minimize pain during needle insertion, ensuring the smooth execution of subsequent injection procedures. Once the anesthesia was administered, the PRP-filled syringe was inserted into the joint cavity through the puncture site. Under ultrasound guidance, the needle path was confirmed to be correct before injecting 3 mL of PRP. Following the injection, the patient was asked to slowly flex and extend the knee joint and remain in the examination room for 10 min of observation. If no adverse reactions occurred, they could resume normal daily activities. Additionally, patients were instructed to keep the puncture site dry and clean for 24 h to prevent infection, and to avoid contact with water at the puncture site and strenuous exercise for 3 days.

The selection principles for single or sequential PRP injection were as follows:

Single PRP injection: (1) Patients with early stage KOA (K-L grade I-II); (2) Patients with mild knee pain, no obvious limitation in knee joint movement, and imaging showing localized cartilage damage; (3) Patients who have shown poor response to conservative treatments or physical therapy but do not require immediate surgical intervention; (4) Patients seeking rapid relief of knee pain symptoms; (5) As the initial phase of a sequential PRP treatment protocol.

Sequential PRP Injection: (1) Patients with moderate-grade KOA (K-L grade II-II) or those who have shown poor response to single PRP treatment; (2) Patients experiencing persistent knee pain with significant mobility limitations, and imaging showing moderate cartilage damage; (3) Patients requiring stimulation for cartilage regeneration, such as the postoperative stage of meniscus or ligament repair surgery; (4) Patients with comorbidities affecting cartilage healing, including diabetes; (5) Patients demonstrating poor PRP response.

The sequential PRP injection group (sequential PRP) received PRP injections every 2 weeks, 3 times in total, at the 0th, 2nd, and 4th weeks, respectively. The single PRP injection group (single PRP) received only one injection at the 0th week.

### Outcome indexes

Laboratory outcomes: blood and synovial fluid samples were collected from patients before treatment and at the 6-month post-treatment for the following analyses: (1) Blood tests: serum interleukin-1β (IL-1β), interleukin-6 (IL-6), and tumor necrosis factor-α (TNF-α) levels. (2) Synovial fluid analysis: a 2 ml knee joint fluid sample was centrifuged, and the supernatant portion was used to detect the levels of matrix metalloproteinase-3 (MMP-3) and tissue inhibitor of metalloproteinases-1 (TIMP-1) through enzyme-linked immunosorbent assay (ELISA).

Clinical outcomes: the following indicators were evaluated before treatment and at 1, 3, and 6 months after treatment: (1) Knee pain severity: visual analog scale (VAS) was used to assess the pain severity, with a score of 0–10 points. The decrease of score indicated the reduction of pain severity. (2) Severity of KOA: the Western Ontario and McMaster Universities Arthritis Index (WOMAC) was used to evaluate the severity of KOA, with a score of 0–96 points (15). The score was positively correlated with the degree of knee joint lesions. (3) Knee joint function: the Lysholm score was used to evaluate the knee joint function, with a score range of 0–100 points (16). The higher score indicated the better function.

Safety outcomes: complications were recorded during the follow-up. Complications related to injection included systemic symptoms such as infection, headache, dizziness and fever, nausea, vomiting, allergy, fatigue, and local symptoms such as redness and pain of knee joint.

### Statistical analysis

SPSS 19.0 software was used for statistical analysis. Quantitative data were expressed in mean ± standard deviation. ANOVA analysis and paired *t*-test were used for inter-group and intra-group comparison of quantitative data, respectively. Inter-group comparison of disordered qualitative data was performed by the *X*^2^ test. Wilcoxon rank sum test and Mann–Whitney rank sum test were used for intra-group and inter-group comparison of ordered qualitative data, respectively. *P* < 0.05 was considered to be a significant difference.

## Results

**Figure 1:**
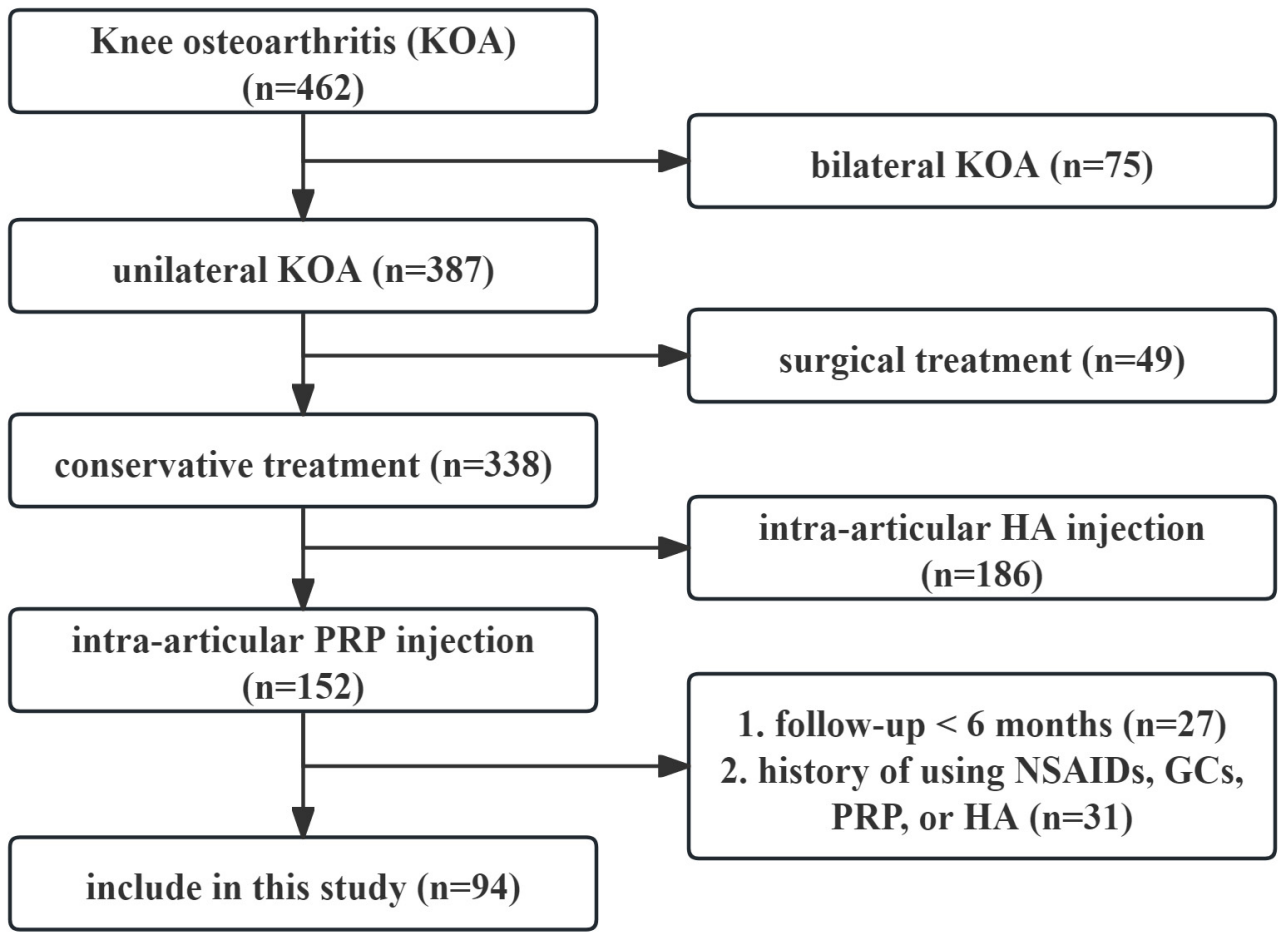
The flow chart of the inclusion and exclusion of patients. KOA, knee osteoarthritis; PRP, platelet-rich plasma, HA, hyaluronate, NSAIDs, nonsteroidal anti-inflammatory drugs, GCs, glucocorticoid.

A total of 94 KOA patients were finally included, including 55 cases in single PRP group and 39 cases in sequential PRP group. The flow chart of the inclusion and exclusion of patients was shown in Figure 1. No significant differences were found in age (*P* = 0.394), gender (*P* = 0.832), body mass index (BMI) (*P* = 0.893), K–L grade (*P* = 0.355) and course of disease (*P* = 0.738) between the two groups (Table 1).

**Table 1:** Baseline data comparison of the two groups.

**Items**	**Single PRP group (*n* = 55)**	**Sequential PRP group (*n* = 39)**	***P*-value**
Age (year), *mean ± SD*	60.05 ± 7.76	58.63 ± 8.16	0.394
**Gender (** ***n*** **)**			0.832
Male	22	17	
Female	33	22	
BMI (kg/m^2^), *mean ± SD*	25.15 ± 3.24	25.06 ± 3.09	0.893
**K-L grade**			0.355
I	5	1	
II	23	16	
III	27	22	
Course of disease (year)	2.50 ± 0.59	2.46 ± 0.54	0.738

**Figure 2:**
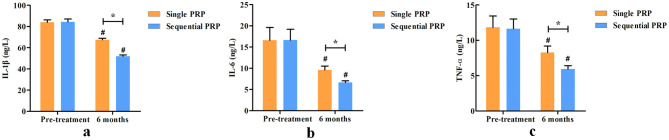
Comparison of serum IL-1β **(a)**, IL-6 **(b)**, and TNF-α **(c)** levels between the two groups. (^#^Intra-group comparison, compared with pre-treatment, *P* < 0.05; *Inter-group comparison, compared with the single PRP group, *P* < 0.05).

There were no significant differences in pre-treatment serum IL-1β, IL-6, and TNF-α between the two groups (*P* = 0.369, 0.365, and 0.571, respectively). At 6-month post-treatment, serum IL-1β, IL-6, and TNF-α were all significantly decreased compared with those pre-treatment in each group (*P* < 0.001 for all the three outcomes), and the sequential PRP group showed lower serum IL-1β, IL-6, and TNF-α than the single PRP group (*P* < 0.001 for all the three outcomes; Figure 2).

**Figure 3:**
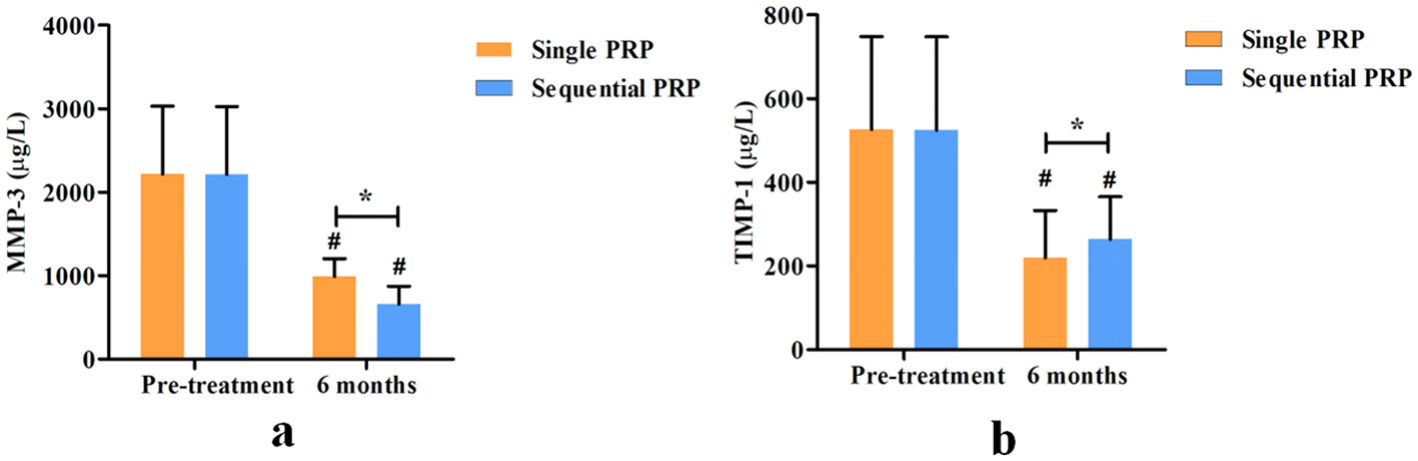
Comparison of synovial fluid MMP-3 **(a)** and TIMP-1 **(b)** levels between the two groups. (^#^Intra-group comparison, compared with pre-treatment, *P* < 0.05; *Inter-group comparison, compared with the single PRP group, *P* < 0.05).

No significant differences were found in pre-treatment synovial fluid MMP-3 and TIMP-1 between the two groups (*P* = 0.250 and 0.417, respectively). At 6-month post-treatment, synovial fluid MMP-3 was significantly decreased (*P* < 0.001) and synovial fluid TIMP-1 was significantly increased (*P* < 0.001) compared with those pre-treatment in each group. Compared with the single PRP group, the sequential PRP group showed a lower synovial fluid MMP-3 (*P* < 0.001) and a higher TIMP-1 (*P* = 0.018) at 6-month post-treatment (Figure 3).

**Figure 4:**
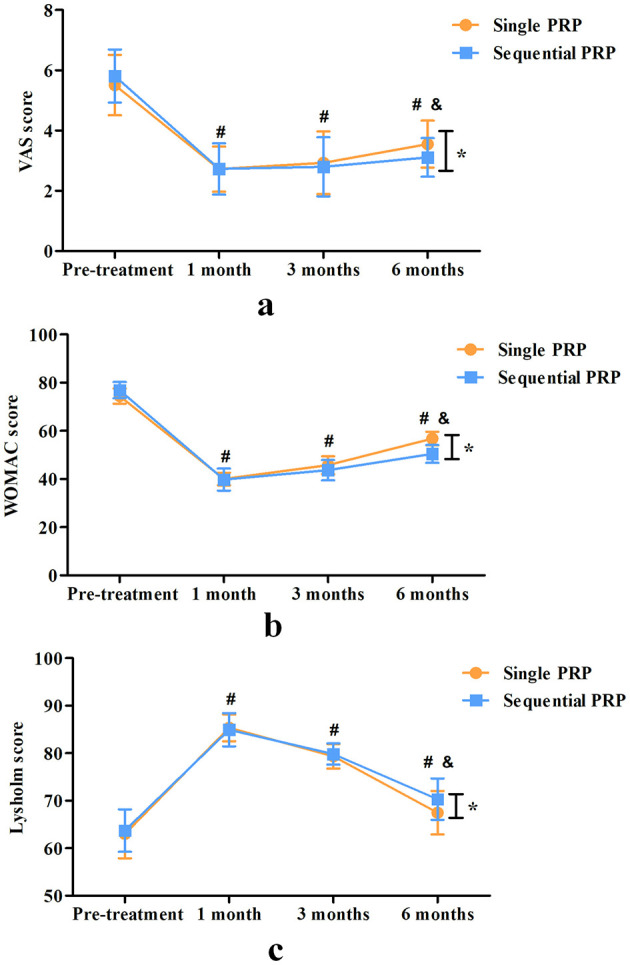
Comparison of VAS score **(a)**, WOMAC score **(b)** and Lysholm score **(c)** between the two groups. (^#^Inter-group comparison, compared with pre-treatment, *P* < 0.05; ^&^Intra-group comparison, compared with 3 months post-treatment, *P* < 0.05, *Inter-group comparison, compared with the single PRP group).

There were no significant differences in VAS score, WOMAC score and Lysholm score between the two groups at pre-treatment, 1 and 3 months post-treatment (VAS score: *P* = 0.149, 0.952, and 0.542, respectively; WOMAC score: *P* = 0.489, 0.789, and 0.893, respectively; Lysholm score: *P* = 0.499, 0.541, and 0.350, respectively). The VAS score, WOMAC score and Lysholm score were all significantly improved in each group at 1 month post-treatment compared with those pre-treatment (*P* < 0.001 for both groups), and all had a certain degree of efficacy loss at 3 and 6 months post-treatment (*P* < 0.001 for both groups). However, compared with single PRP group, sequential PRP had a lower VAS score and WOMAC score, and a higher Lysholm score at 6 months post-treatment (*P* = 0.005, *P* < 0.001, and *P* < 0.001, respectively; Figure 4).

A total of five patients had complications in sequential PRP group, including four cases of knee joint redness and one case of transient fever. While three patients of complications were found in single PRP group, including one cases of knee joint redness and two cases of knee joint pain. No significant difference was found in complications rate (12.82% vs 5.45%) between the two groups (*P* = 0.269) and all cases were cured after active treatment.

## Discussion

KOA is a degenerative disease characterized by pain and deformity, resulting from various etiological factors that lead to articular cartilage fibrosis, rupture, detachment, ulceration, and even cartilage loss (2). The destruction of chondrocytes and the cartilage matrix generates substantial metabolites, and the accumulation of type II collagen carboxyl-termini and chondrofibrillar proteins in the synovial cavity promotes the production and activation of inflammatory cytokines and related degrading enzymes, thereby exacerbating cartilage destruction and disrupting the equilibrium between progressive cartilage damage and extracellular matrix formation (2, 9). It has also been revealed that the development of KOA is closely associated with multiple cytokines and proteins (17). Notably, matrix metalloproteinases (MMPs) break down the extracellular matrix of articular cartilage, leading to thinning of the cartilage layer and reduced resistance to external stimuli, which further aggravates cartilage damage (18). Additionally, inflammatory factors also play a significant role in the progression of KOA (19).

MMP-3 directly damages the articular cartilage matrix by degrading type II collagen and proteoglycans. As a key enzyme in the cartilage degeneration associated with OA and rheumatoid arthritis (RA), MMP-3 activity correlates positively with the severity of articular cartilage damage, making it a biomarker for disease progression (20). TIMP-1 (tissue inhibitor of metalloproteinases-1) specifically inhibits MMP-3 and other matrix metalloproteinases to slow cartilage degradation. Imbalances in TIMP-1 levels (e.g., an elevated MMP-3/TIMP-1 ratio) exacerbate joint destruction (21). Additionally, TIMP-1 participates in cellular signaling and influences the inflammatory microenvironment (22). The dynamic balance between MMP-3 and TIMP-1 determines the direction of cartilage metabolism, and monitoring their levels helps assess arthritis severity and treatment efficacy.

Interleukins (ILs), cytokines secreted by leukocytes or immune cells, play core roles in immune regulation, inflammation, and hematopoietic functions. IL-1β, a pro-inflammatory factor secreted by macrophages and chondrocytes, activates NF-κB pathways to promote MMP expression, directly degrading collagen and proteoglycans, leading to cartilage destruction (23). In KOA patients, serum and synovial fluid levels of IL-1β are significantly elevated, showing a positive correlation with disease severity (24). IL-6, produced by synovial fibroblasts and chondrocytes, amplifies inflammatory responses through JAK-STAT pathways while inducing acute-phase protein synthesis (e.g., CRP), participating in subchondral bone remodeling (25). Studies demonstrate that IL-6 levels in synovial fluid of KOA patients are positively correlated with joint narrowing and osteophyte accumulation (26). TNF-α is a core pro-inflammatory factor in arthritis (especially rheumatoid arthritis). It drives the inflammatory cascade by activating the NF-κB pathway, promotes cartilage destruction and bone resorption, and is a key target for targeted therapy (27).

This study found that administering PRP therapy to early/mid stage KOA patients via single or sequential injections resulted in a significant reduction of serum inflammatory markers (including IL-1β, IL-6, and TNF-α levels). Synovial fluid analysis revealed markedly decreased MMP-3 levels and substantially increased TIMP-1 levels. These results demonstrated that both single and sequential PRP injections can effectively regulate inflammation and cytokine levels in KOA patients (28). The study findings also indicated that at 1, 3, and 6 months post-treatment, both groups showed significant improvements in VAS, WOMAC, and Lysholm scores compared to pre-treatment levels. Therefore, we confirmed that intra-articular injection of PRP can achieve satisfactory clinical efficacy in the treatment of KOA (29). However, at the 6-month follow-up, the single PRP group had lower VAS and WOMAC scores than the sequential PRP group, while the Lysholm score remained more favorable in the sequential PRP group. This suggested that single PRP treatments lack sustained long-term efficacy, with diminishing effects on knee pain and function improvement over time. Conversely, sequential PRP injections consistently yielded improved knee symptoms and functional outcomes, demonstrating excellent medium-to-long term therapeutic benefits. Therefore, for intra-articular PRP injection therapy, sequential treatments outperformed single-dose administration in effectively alleviating pain and enhancing joint function, offering superior efficacy and stability. No significant difference was found in complications between the two groups, and all complications cases were cured after active treatment, indicating the safety of the two PRP injection methods in KOA treatment. However, redness and pain in the knee joints were not uncommon in both groups. This indicated that during knee joint injections, close attention must be paid to aseptic operation and precise technique (such as puncture under ultrasound guidance). Additionally, measures such as applying ice packs within 48 h after injection to relieve swelling and using crutches or braces to assist walking to reduce joint load may also be beneficial.

This study had several limitations: first, the patient sample size was relatively small. Second, the short follow-up period made it impossible to evaluate long-term outcomes of sequential PRP treatments. Third, the assessment criteria were primarily subjective, lacking objective comparisons indicators such as knee MRI scans.

In conclusion, both sequential and single injection of PRP can achieve satisfactory clinical efficacy in the treatment of early/mid-stage unilateral KOA, but sequential intra-articular PRP injection has the advantages of sustained long-term efficacy in relieving pain and improving function of knee joint.

## Data Availability

The data analyzed in this study is subject to the following licenses/restrictions: Data will be made available on request. Requests to access these datasets should be directed to Xiuli Wei, 13971928700@163.com.
